# Transcriptome analysis of *Gossypium hirsutum* cultivar Zhongzhimian No.2 uncovers the gene regulatory networks involved in defense against *Verticillium dahliae*

**DOI:** 10.1186/s12870-024-05165-7

**Published:** 2024-05-27

**Authors:** Xi-Yue Ma, Xiao-Han Zhou, Bin-Bin Liu, Ye-Jing Zhang, He Zhu, Yue Li, Zi-Sheng Wang, Xiao-Feng Dai, Jie-Yin Chen, Zhen-Qi Su, Ran Li

**Affiliations:** 1 State Key Laboratory for Biology of Plant Diseases and Insect Pests, Institute of Plant Protection, Chinese Academy of Agricultural Sciences, Beijing, 100193 P.R. China; 2 College of Agronomy and Biotechnology, China Agricultural University, Beijing, 100193 P.R. China; 3 Western Agricultural Research Center, Chinese Academy of Agricultural Sciences, Changji, 831100 China; 4 The Cotton Research Center of Liaoning Academy of Agricultural Sciences, Liaoning Provincial Institute of Economic Crops, Liaoyang, 111000 China

**Keywords:** Verticillium wilt, *G. hirsutum* cultivar Zhongzhimian No.2, Resistance, Transcriptome sequencing, Oxidation-reduction process

## Abstract

**Background:**

Cotton is globally important crop. *Verticillium* wilt (VW), caused by *Verticillium dahliae*, is the most destructive disease in cotton, reducing yield and fiber quality by over 50% of cotton acreage. Breeding resistant cotton cultivars has proven to be an efficient strategy for improving the resistance of cotton to *V. dahliae*. However, the lack of understanding of the genetic basis of VW resistance may hinder the progress in deploying elite cultivars with proven resistance.

**Results:**

We planted the VW-resistant *Gossypium hirsutum* cultivar Zhongzhimian No.2 (ZZM2) in an artificial greenhouse and disease nursery. ZZM2 cotton was subsequently subjected to transcriptome sequencing after Vd991 inoculation (6, 12, 24, 48, and 72 h post-inoculation). Several differentially expressed genes (DEGs) were identified in response to *V. dahliae* infection, mainly involved in resistance processes, such as flavonoid and terpenoid quinone biosynthesis, plant hormone signaling, MAPK signaling, phenylpropanoid biosynthesis, and pyruvate metabolism. Compared to the susceptible cultivar Junmian No.1 (J1), oxidoreductase activity and reactive oxygen species (ROS) production were significantly increased in ZZM2. Furthermore, gene silencing of *cytochrome c oxidase subunit 1* (*COX1*), which is involved in the oxidation-reduction process in ZZM2, compromised its resistance to *V. dahliae*, suggesting that *COX1* contributes to VW resistance in ZZM2.

**Conclusions:**

Our data demonstrate that the *G. hirsutum* cultivar ZZM2 responds to *V. dahliae* inoculation through resistance-related processes, especially the oxidation-reduction process. This enhances our understanding of the mechanisms regulating the ZZM2 defense against VW.

**Supplementary Information:**

The online version contains supplementary material available at 10.1186/s12870-024-05165-7.

## Background

Cotton is one of the most important economic crops worldwide for the significant economic value of its textile fiber, feed, foodstuff, oil, and biofuel products. VW caused by *V. dahliae* is the most devastating vascular disease of cotton, which can affect more than 50% of cotton acreage and significantly reduces yield and fiber quality [1]. It is difficult to control this pathogen with fungicides because *V. dahliae* resides in vascular tissues and is transmitted in cotton plants [2]. Breeding resistant cotton cultivars is considered an optimal method to prevent and control VW. However, it is challenging to produce VW-resistant cotton germplasm by improving genetic resistance because of the complex resistance mechanism of cotton to VW. For instance, terpenoid aldehydes and phenylpropanoids, reactive oxygen species, salicylic acid, jasmonic acid, ethylene, and brassinosteroids signaling pathways are involved in cotton resistance to VW [3, 4]. Therefore, it is crucial to reveal the genetic basis of VW resistance in cotton germplasm. With the rapid development of next-generation sequencing, the high-quality diploid and allotetraploid *Gossypium* species genomes were assembled, improving our understanding of cotton properties and facilitating efficient analysis by associating mapping [5]. In addition, a large number of genes annotated from the cotton genome, which were involved in resistance against VW, have been identified via biotechnology, various omics, high-throughput sequencing technologies, and genome-wide association studies (GWAS). For instance, lysin motif (LysM)-containing proteins were identified from diploid *G. raimondii* and *G. arboreum*, tetraploid *G. hirsutum* acc. TM-1, and *G. barbadense* acc. 3–79, respectively [6]; ascorbate peroxidase members mediated oxidoreductive metabolism for VW resistance was uncovered by comparative proteomic analysis [7]; single nucleotide polymorphisms (SNPs) related to cotton VW resistance were detected using specific-locus amplified fragment sequencing (SLAF-seq) and the locus of CG02 was detected as an important gene for resistance against *V. dahliae* in cotton [8]; L-type lectin-domain containing receptor kinase (GhLecRKs-V.9) and non-specific lipid transfer protein GhnsLTPsA10 were identified to play important roles in VW resistance via GWAS [9, 10]; and hundreds of microRNAs (miRNAs) were identified and miR477 regulated VW resistance in cotton by directly cleaving *GhCBP60A* [11].

Plants are often exposed to various pathogens in the course of their existence, and they possess efficient defense mechanisms to protect themselves from disease. Pathogen-associated molecular patterns (PAMP)-triggered immunity (PTI) and effector-triggered immunity (ETI) defense responses are triggered by different pathogenic molecules. PTI is the first response based on the recognition of PAMPs leading to the production of ROS, the activation of mitogen-activated protein kinases (MAPKs), the deposition of callose in the cell wall, and the expression of pathogenesis-related proteins [12–15]. ETI is triggered by effectors secreted from pathogens which are recognized by the components of immune mechanisms in the host, such as nucleotide-binding leucine-rich repeat (NB-LRR) proteins, and this recognition activates the plant immune response [16]. Therefore, identifying plant resistance genes provides a basis for not only investigating disease resistance mechanisms but also deploying candidate genes to develop disease-resistant crop varieties. Several genes have been identified that contribute to cotton defense response against VW, and the functional mechanism has been revealed. For example, acetylation of Calmodulin protein 7 GhCaM7 could enhance the cotton resistance to *V. dahliae* [17]; 4-coumarate-CoA ligase 3 (*Gh4CL3*) acts as a positive regulator for cotton resistance against *V. dahliae* by promoting jasmonic acid (JA) signaling mediated enhanced cell wall rigidity and metabolic flux [18]; transmembrane protein 214 GbTMEM214s are a kind of transmembrane protein which is induced by *V. dahliae* inoculation, and *GbTMEM214s*-silenced lines significantly decreased the resistance to VW [19]; Oxo-phytodienoic acid reductase GhOPR9 interacted with sucrose galactosyltransferase GhRFS6 and regulated cotton resistance to VW through the regulation of the JA pathway [20, 21]; germin-like protein GhABP19 played important roles in the regulation of resistance to VW by exerting SOD activity and its ability to activate the JA pathway [22]; U-box E3 ligase GhPUB17 was inhibited by antifungal protein GhCyP3 with antifungal activity and served as a negative regulator involved in cotton resistance to *V. dahilae* [23]; transcription factor *GhMYB4* acted as a negative regulator in lignin biosynthesis resulting in alteration of cell wall integrity and activation of cotton defense response [24].

Although a large number of VW-resistance-related genes have been discovered and their resistance mechanisms in cotton have been identified, the mechanism by which cotton cultivars are resistant is still poorly understood, including *G. hirsutum* cultivar Zhongzhimian No.2 (ZZM2). ZZM2 exhibits outstanding characteristics including cotton bollworm resistance, *Fusarium* wilt resistance, VW resistance, high yield, good stability, adaptability, and is the most widely planted VW-resistant cultivar in China [1, 25]. However, the mechanism underlying this resistance to VW has not been clearly defined. Previously, we sequenced the entire genome of ZZM2 and predicted the function of secreted proteins in ZZM2 in disease resistance against VW [26]. In the present study, we validated the ZZM2 resistance to VW of and conducted transcriptome sequencing to identify the genes expressed by ZZM2 in response to *V. dahliae* strain Vd991, thereby elucidating the regulatory gene networks involved in defense against VW. Several differentially expressed genes (DEGs) were identified in response to *V. dahliae* inoculation at 6, 12, 24, 48, and 72 h post-inoculation (hpi). Functional enrichment of DEGs was strongly associated with resistance-related functions, including flavonoid and terpenoid quinone biosynthesis, plant hormone signaling, MAPK signaling, phenylpropanoid biosynthesis, and pyruvate metabolism. Furthermore, the expression of DEGs in ZZM2 was enriched in oxidoreductase activity, in contrast to that in the susceptible cultivar J1. Reactive oxygen species (ROS) production in ZZM2 was significantly higher than that in J1. Furthermore, gene silencing of *cytochrome c oxidase subunit 1* (*COX1*) is involved in the oxidation-reduction process in ZZM2, which displays increased susceptibility to *V. dahliae*, suggesting that *COX1* confers VW resistance in ZZM2. Our findings demonstrate that the *G. hirsutum* cultivar ZZM2 responds to *V. dahliae* via resistance-related processes, notably the oxidation-reduction, thereby enhancing our understanding of response mechanisms to *V. dahliae*.

## Materials and methods

### Plant growth conditions and fungal pathogen inoculations

The resistant *G. hirsutum* cultivar (cv.) Zhongzhimian No.2 (ZZM2) and susceptible *G. hirsutum* cultivar (cv.) Junmian No.1 (J1) was grown and maintained in a greenhouse at 28 ℃ under 16 h light/8 h dark photoperiod. Moreover, ZZM2 and J1 were planted at the disease nursery in Xinxiang, located in central of China (35^o^18’ north latitude, 113^o^52’ east longitude). 3-weeks-old cotton was used for *V. dahliae* inoculation. The highly virulent *V. dahliae* strain Vd991 was cultured and adjusted to 10^7^ conidia/ml for inoculating cotton seedlings. Roots of the seedlings were immersed in the conidial suspension for 10 min and then planted into soil [26].

### Pathogenicity assays

Pathogenicity was assessed 3 weeks after inoculation, and vascular discoloration in shoots was assessed visually at 4 weeks after inoculation. Twenty cotton seedlings were inoculated for pathogenicity accession, and three replicates were detected for pathogenicity assay. The statistics comprising of five grades (0, 1, 2, 3 and 4) were used for assigning infected plants. Grade 0 indicates that the plant is healthy and has no disease symptoms, grade 1 to grade 4 represent the typical yellowing and wilting observed in 0–25%, 25-50%, 50-75% and 75-100% of leaves investigated plants respectively. The disease index (DI) of *Verticillium* wilt was calculated according to the following formula:

$$\text{D}\text{I}=\frac{\sum (\text{n}\hspace{0.17em}\times \hspace{0.17em}\hspace{0.17em}\text{n}\text{u}\text{m}\text{b}\text{e}\text{r}\hspace{0.17em}\text{o}\text{f}\hspace{0.17em}\text{p}\text{l}\text{a}\text{n}\text{t}\text{s}\hspace{0.17em}\text{a}\text{t}\hspace{0.17em}\text{l}\text{e}\text{v}\text{e}\text{l} \text{n})}{4\hspace{0.17em}\times \hspace{0.17em}\hspace{0.17em}\text{t}\text{h}\text{e}\hspace{0.17em}\text{n}\text{u}\text{m}\text{b}\text{e}\text{r}\hspace{0.17em}\text{o}\text{f}\hspace{0.17em}\text{t}\text{o}\text{t}\text{a}\text{l}\hspace{0.17em}\text{p}\text{l}\text{a}\text{n}\text{t}\text{s}\times 100}$$

Fungal biomass quantification was performed by amplification of *V. dahliae* elongation factor *VdEF-1α* normalized by the cotton *GhUbiquitin* gene through quantitative PCR (qPCR). Primers are listed in Table S1.

### *Verticillium Dahliae* recovery assay

Stem fragments of 2 cm were sectioned from the first node of the stem base to the one above for both ZZM2 and J1 at 14 days post inoculation (dpi) [27]. The surfaces of stem fragment were sterilized with 70% ethanol for 20–30 s and then immersed in 0.1% corrosive sublimate (HgCl_2_ solution) for 1 min. After that stem fragments were washed with 0.1% HgCl_2_ solution (one time) and with sterilized distilled water (five times). From sterilized stem 3–4 mm sized sliced were cut in cross sectional, placed on PDA medium and incubated in dark at 25 ℃ for 5 days. Plant susceptibility to infection was defined according to the number of stem sections from which the fungus grew. Twenty cotton seedlings were inoculated for pathogenicity accession, and three replicates were detected for pathogenicity assay.

### RNA extraction, library construction and RNA sequencing analysis

The root of 3-weeks-old seedlings of the resistance cotton cv. ZZM2 was dipped in 10^7^ conidia/ml suspension of Vd991 for 10 min and then planted into soil. The roots were sampled at 6 h post inoculation (hpi), 12 hpi, 24 hpi, 48 hpi and 72 hpi, and the roots without inoculation were sampled as 0 hpi. All samples were immediately frozen in liquid nitrogen and stored at -80 ℃ until further use. Total RNA samples were extracted using an RNA Purification Kit (Tiangen, Beijing, China) and prepared for sequencing with three biological replicates for each sample. Genomic DNA was removed by DNase treatment and rRNA was removed by Ribo-zeroTM rRNA Removal Kit (Epicenter, USA). Strand-specific sequencing was performed on an Illumina HiSeq X-Ten by BGI (BGI-genomics, Shenzhen), which generated 125 bp paired-end reads. Raw data were processed through in-house perl scripts to obtain clean reads. The clean reads were obtained by removing the adapter and low-quality reads (quality score > Q20). Over 15 GB of clean data were generated from each sample. The clean reads were mapped onto the reference genome of *G. hirsutum* cv. Zhongzhimian No.2 (GenBank: JAMQUR000000000) using Tophat2 (v2.0.9) [28] and Bowtie 2 (v2.2.9) [29] (Table S2).

### Identification of differentially expressed genes (DEGs)

A total of six groups were selected for sequencing, including Vd991 inoculated cv. ZZM2 at 6 hpi, 12 hpi, 24 hpi, 48 hpi, and 72 hpi as the treatment group and non-inoculated (0 hpi) as the control. Fragments Per Kilobase of the transcript per Million mapped reads (FPKM) was used to determine expression values. Cuffdiff (v2.1.1) was used to calculate the FPKM of genes in each sample [30]. The fold-change in gene expression value was calculated by FPKM treat/FPKM control. Transcripts were identified as differentially expressed (DEGs) between treatment and control with parameters of fold change > 2 and a p-value < 0.05 (Table S3).

### Functional annotation

The DEGs were analyzed using Gene Ontology (GO) analysis by WEGO website database and Kyoto Encyclopedia of Genes and Genomes (KEGG) analysis [31] using the KEGG Orthology (KO)-Based Annotation System (KOBAS) to explore their biological roles. GO analysis was in three terms, including cellular component, molecular functional and biological function with the significant enrichment tested by the Pearson chi-square test (*P* < 0.05) from the WEGO tool.

### Reverse transcription and quantitative PCR (RT-qPCR)

The seedlings of resistant cv. Zhongzhimian No.2 and susceptible cv. Junmian No.1 were inoculated with Vd991 as described above. The roots of cotton at 6 hpi, 12 hpi, 24 hpi, 48 hpi, 72 hpi, and non-inoculated were collected for RNA extraction. RNA aliquots of 2 µg were used for cDNA synthesis by the TranScript One-Step gDNA Removal and cDNA Synthesis SuperMix kit (Trans, Beijing, China). Quantitative PCR (qPCR) was performed using a qPCR SYBR premix Ex TaqII kit (TaKaRa, Tokyo, Japan). The relative quantification of RT-qPCR was measured by 2^−∆∆Ct^ analysis method. The mRNA expression levels were normalized using cotton gene *GhUbiquitin*. Three biological replicates were performed for each experiment, with three technical replicates. The genes related to the redox process were detected. The specific primers used are listed in Table S1.

### Measurement of H_2_O_2_ content and detection of ROS accumulation

3-weeks-old cotton was used for inoculation with Vd991, 5 × 10^6^ conidia/ml. The content of H_2_O_2_ was detected in cotton roots at 3 dpi, 5 dpi, and 7 dpi, respectively, using a Micro Hydrogen Peroxide Assay Kit (BC3590, Solarbio, Beijing, China), described by Lu et al. [7]. The roots without Vd991 inoculation (0 dpi) were used as the control. ROS accumulation was detected in cotton stems at 7 dpi and 14 dpi using 303-diaminobenzidine (DAB) solution as described by Li et al. [32]. The stems treated with sterile water were used as the mock. Twenty cotton seedlings were inoculated for pathogenicity accession, and three replicates were detected for pathogenicity assay.

### Virus-induced gene silencing (VIGS) assays in cotton

For the VIGS assays, approximately 500 bp fragments of *COX1* were amplified from cv. ZZM2 genomic DNA [33]. Fragments were separately integrated into pTRV2 vector and introduced into *A. tumefaciens* GV3101. *Agrobacterium* strains harboring the recombinant plasmid were combined with strains harboring the pTRV1 vector in a 1:1 ratio and co-infiltrated into cotyledons of two-week-old cv. ZZM2 seedlings [33]. The silencing efficiency for *COX1* was determined by RT-qPCR, which compared gene expression in TRV2:COX1-infiltration plants with gene expression in TRV2:00-infiltration plants. The expression of *COX1* was normalized using cotton gene *GhUbiquitin*. Primers are listed in Table S1.

### Statistical analysis

Results of gene expression and fungal biomass assays were analyzed using SPSS (version 20.0) software. Significant differences were detected by pairwise *t*-test and shown by the probabilities associated with the test (* indicated *P* < 0.05, ** indicated *P* < 0.01).

## Results

### *G. hirsutum* cultivar ZZN2 exhibits VW resistance

**Fig. 1:**
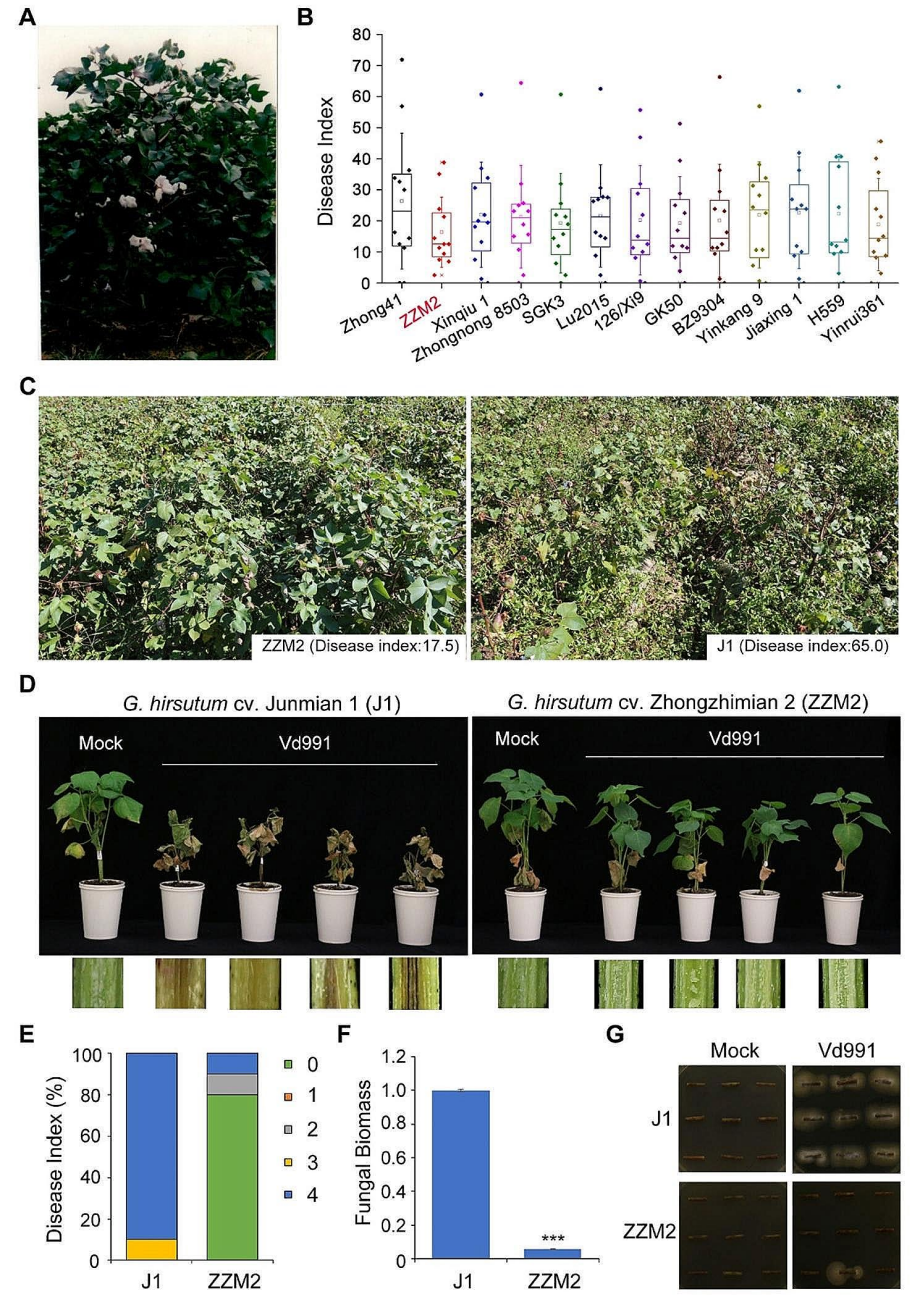
Characteristic analysis of *G. hirsutum* cv. Zhongzhimian No.2 (ZZM2) in Verticillium wilt resistance. **(A)** Phenotype of ZZM2 at the boll opening stage. (**B**) Disease index of Verticillium wilt among 13 varieties experienced in Yellow River basin in 2005. (**C**) Disease symptoms of resistant ZZM2 and susceptible *G. hirsutum* cultivar Junmian No.1 (J1) in disease nursery of Liaoyang city at July 2023. (**D**) Disease symptoms and stem vascular discoloration of ZZM2 and J1 cotton following inoculation with *Verticillium dahliae* strain Vd991 and treatment with water as mock/control. The severity of cotton wilting as shown in the corresponding pictures recorded at 14 days post inoculation (dpi). (**E**) Disease index of ZZM2 and J1 cotton were determined at 14 dpi after Vd991 inoculation. Disease grade 0, 1, 2, 3, and 4 showed the disease range from asymptomatic to lethal. (**F**) Fungal biomass of ZZM2 and J1 was detected by quantitative PCR analysis. 15 inoculated plants were used for detection and the Verticillium *EF-1α* gene was used as a reference gene. (**G**) *V. dahliae* recovery assay. Number of stem sections from which fungus grew described extent of fungal colonization. Photos were taken at 7 days after plating. Three biological replicates were conducted for analysis

*G. hirsutum* cultivar ZZM2 was bred by introducing Bt genes into the cotton cultivar Zhongzhi 372 and showed characteristics of cotton bollworm resistance, *Fusarium* wilt resistance, VW resistance, high yield, good yield stability, and adaptability. The ZZM2 plants were high, their branches were long and flat, their stems were thick and strong, and they were full of fluff. The leaves were medium in size and relatively flat, and the bolls were comparatively large, nearly round, and easily opened to discharge batting (Fig. 1A). Regional experiments were conducted in the Yellow River Basin in 2005 and involved several cotton varieties, along with ZZM2. The disease index of ZZM2 was lower than that of other cultivars (Fig. 1B). To verify the resistance of ZZM2 to *V. dahliae*, disease symptoms were confirmed in a disease nursery in Liaoyang in 2022. The disease index of ZZM2 was 17.5, and the disease index of susceptible *G. hirsutum* cultivar J1 was 65.0, thus ZZM2 showed a significantly resistant phenotype compared with J1 (Fig. 1C). In addition, seedlings of both ZZM2 and J1 cultivars planted in a greenhouse for three weeks were inoculated with Vd991. ZZM2 seedlings showed a highly resistant phenotype against Vd991 compared to J1 21 days post-inoculation (Fig. 1D). The disease index of ZZM2 was lower than that of J1 (Fig. 1E), and fungal biomass of ZZM2 was relatively lower than that of J1 which is nearly 90% (Fig. 1F). Colonies thrived from ZZM2 stems, while there was minimal growth observed from J1 stems in fungal recovery assays (Fig. 1G). Thus, the assays revealed that fewer fragments of ZZM2 were infected with Vd991 compared to J1. Overall, ZZM2 is a cotton cultivar that has resistance to *V. dahliae* infection.

### Identification of DEGs in ZZM2 during *V. dahliae* infection by RNA-seq

**Fig. 2:**
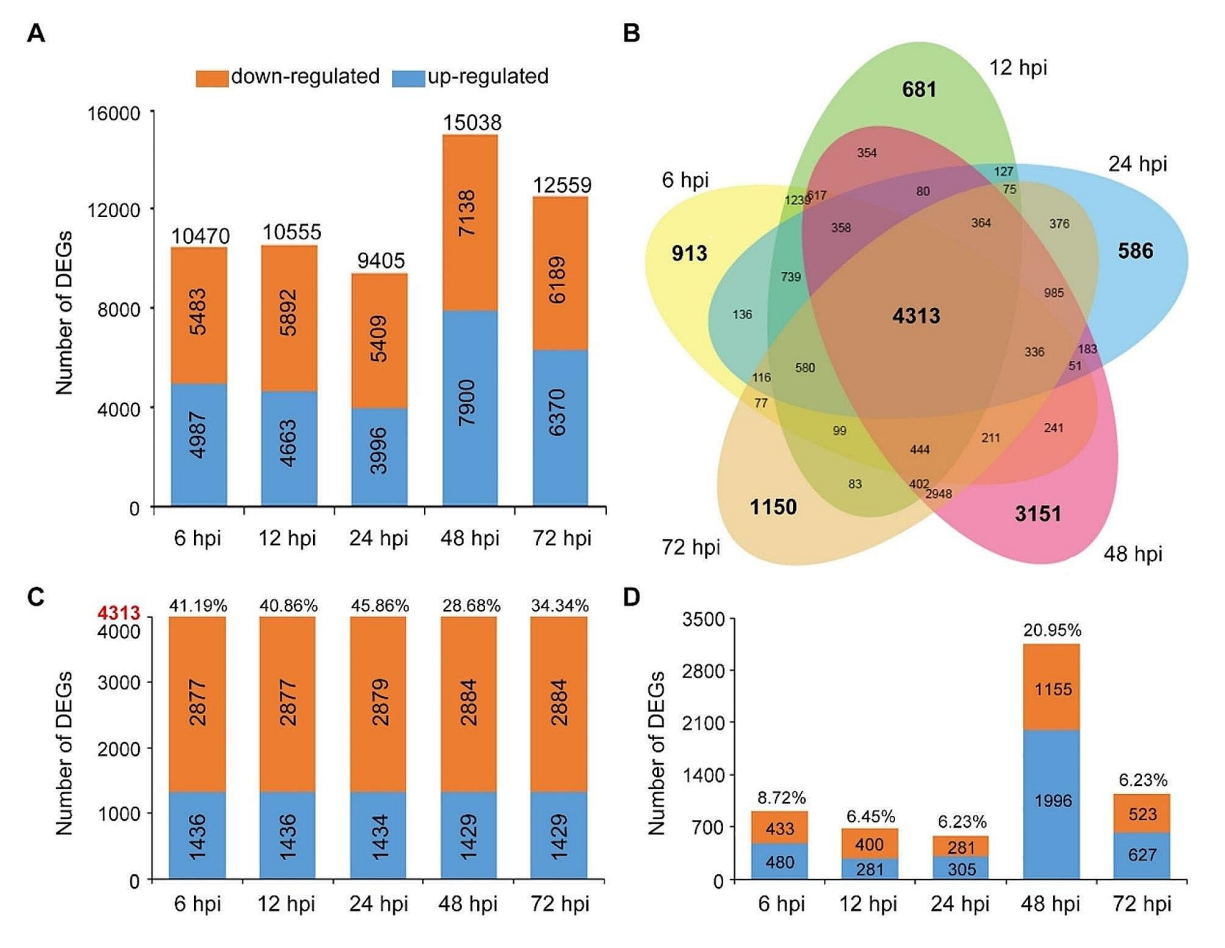
Analysis of differentially expressed genes (DEGs) in *G. hirsutum* cv. Zhongzhimian No.2 (ZZM2) during *Verticillium dahliae* inoculation. (**A**) Statistic of the number of DEGs which up- and down-regulated at 6 hpi, 12 hpi, 24 hpi, 48 hpi, and 72 hpi after *V. dahliae* inoculation. (**B**) The Venn diagram of the DEGs indicated unique and common DEGs in stage of 6 hpi, 12 hpi, 24 hpi, 48 hpi, and 72 hpi. (**C**) Statistic of 4313 co-expressed DEGs among five comparisons. The number showed the number of up- and down-regulated DEGs respectively, and the percentage indicated the proportion of these 4313 co-expressed DEGs to whole DEGs at correspondent time stage. (**D**) Statistic of specific DEGs at five comparisons respectively. The number showed the number of up- and down-regulated DEGs respectively, and the percentage indicated the proportion of these 4313 co-expressed DEGs to whole DEGs at correspondent time stage

As the cotton cultivar ZZM2 showed a significant *V. dahliae*-resistant phenotype, RNA-seq was performed to identify the genetic network in the response to *V. dahliae* infection. Seedlings of ZZM2 were inoculated with *V. dahliae* isolate Vd991, and root samples were collected at 0 hpi (without inoculation), 6 hpi, 12 hpi, 24 hpi, 48 hpi, and 72 hpi. DEGs were identified at 6, 12, 24, 48, and 72 hpi and compared to those at 0 h. Thousands of DEGs were identified at each stage (Fig. 2A). The number of up-regulated DEGs was lower than that of down-regulated DEGs at 6, 12, and 24 hpi, whereas the number of up-regulated DEGs was higher than that of down-regulated DEGs at 48 and 72 hpi (Fig. 2A). The number of DEGs at 48 hpi was the highest among all stages, with 15,038 genes responsive to *V. dahliae* infection (Fig. 2A). Moreover, a Venn diagram illustrates that 4313 DEGs continually responded to *V. dahliae* infection, whereas 913, 681, 586, 3151, and 1150 DEGs were differentially expressed at 6, 12, 24, 48, and 72 hpi, respectively (Fig. 2B). Among 4313 DEGs, the number of down-regulated DEGs was approximately twice as many as the number of up-regulated DEGs, and 4313 DEGs accounted for 28.68% of all DEGs identified at 48 hpi, which was the lowest percentage among all stages (Fig. 2C). In addition, the number of specific DEGs at each stage was lower than that of constitutively expressed DEGs, accounting for 10% of the number of DEGs at the corresponding stage (Fig. 2D). 3151 genes were specifically differentially expressed at 48 hpi, with 20.95% of all DEGs identified at 48 hpi (Fig. 2D). Therefore, many genes were differentially expressed in response to *V. dahliae* infection. These genes were continually and uniquely expressed during *V. dahliae* infection, and 48 hpi after *V. dahliae* infection is an important stage at which ZZM2 responds to *V. dahliae*.

### Functional analysis of DEGs in ZZM2 response to *V. dahliae* infection

**Fig. 3:**
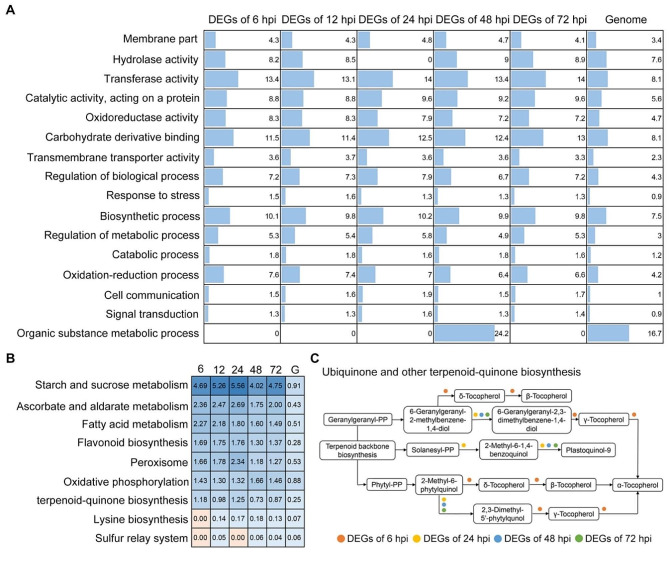
Functional annotation of the DEGs in cotton cv. Zhongzhimian No.2 (ZZM2) during *Verticillium dahliae* inoculation. (**A**) Gene ontology (GO) annotation predicted DEGs at 6 hpi, 12 hpi, 24 hpi, 48 hpi, and 72 hpi respectively versus the predicted protein-coding genes from the whole genome. Columns represented the percentage of enriched in the items, and the significant enrichment was determined by a Pearson chi-square test at *P* < 0.05. (**B**) The potential pathways were predicted by the KEGG using DEGs at 6 hpi, 12 hpi, 24 hpi, 48 hpi, and 72 hpi respectively compared with the predicted protein-coding genes from the whole genome. Numbers represented the percentage of enriched in the pathway. (**C**) Enrichment of DEGs in the pathway of ubiquinone and other terpenoid-quinone biosynthesis

The DEGs expressed in response to *V. dahliae* infection were related to several biological processes, such as stress response. The functional characteristics of DEGs identified at 6, 12, 24, 48, and 72 hpi were predicted using GO and KEGG. A total of 73 categories were enriched according to GO annotation, including kinase activity, regulation of biological and metabolic processes, and stress response (*P* < 0.05) (Table S4). Moreover, 16 categories were significantly enriched in DEGs responses to *V. dahliae* infection (Fig. 3A). A portion of the DEGs was relevant to resistance, as previously reported, such as genes for oxidoreductase activity, response to stress, and oxidation-reduction processes (Fig. 3A). In addition, KEGG network analysis showed that 137 pathways were matched by DEGs response to *V. dahliae* infection (Table S5). Among these pathways, several DEGs were highly enriched for starch and sucrose metabolism, ascorbate and aldarate metabolism, flavonoid biosynthesis, peroxisomes, and terpenoid-quinone biosynthesis (Fig. 3B). Terpenoid quinone is a vital compound involved in the stress response of plants. Thus, the ubiquinone and other terpenoid-quinone biosynthesis pathways were further predicted by DEGs at each stage after *V. dahliae* inoculation. The results showed that synthesis of α-, β-, γ-, and δ-tocopherol was only matched by DEGs at 6 hpi, and production of 2-methyl-6-1,4-benzoquinol was only related to DEGs at 24 hpi (Fig. 3C), while synthesis of 6-geranylgeranyl-2,3-dimethylbenzene-1,4-diol, plastoquinol-9, and 2,3-dimethyl-5’-phytylquinol genes were differentially expressed at 24 hpi, 48 hpi, and 72 hpi instead of 6 hpi (Fig. 3C). Taken together, the function of the gene that respond to *V. dahliae* inoculation coordinates with the response to stress, redox reactions, and biosynthesis of resistance-related compounds.

### Functional analysis of continuously expressed DEGs in ZZM2 response to *V. dahliae* infection

**Fig. 4:**
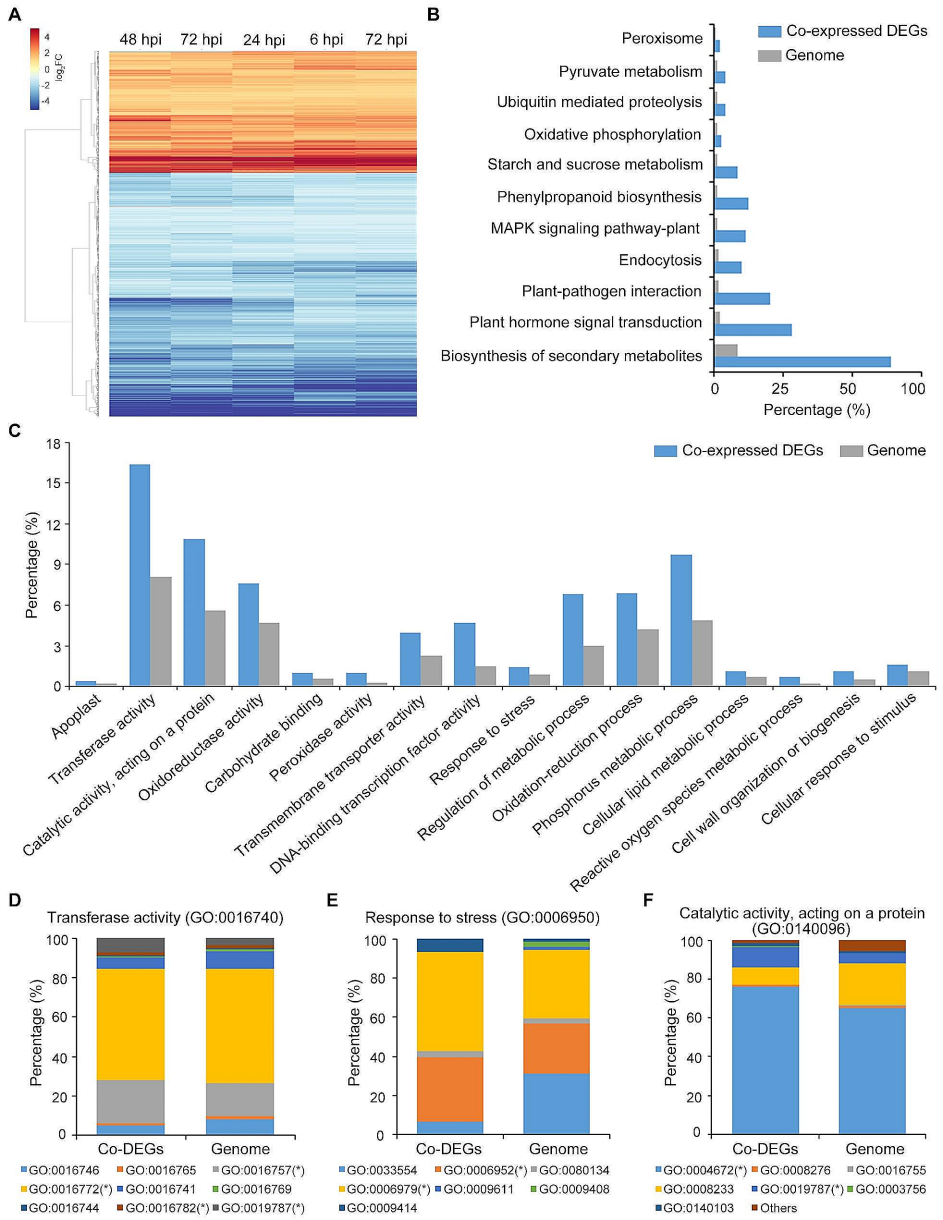
Functional annotation of the co-expressed DEGs in cotton cv. Zhongzhimian No.2 (ZZM2) during *Verticillium dahliae* inoculation. (**A**) Heatmap represented the time course expression profiles of 4313 co-expressed DEGs annotated in ZZM2 at 6 hpi, 12 hpi, 24 hpi, 48 hpi, and 72 hpi, which was performed by FPKM values of all genes. **(B)** The potential pathways were predicted by the KEGG database 4313 co-expressed DEGs versus the predicted protein-coding genes from the whole genome. **(C)** Gene ontology (GO) annotation predicted 4313 co-expressed DEGs versus the predicted protein-coding genes from the whole genome, and significant enrichment was determined by a Pearson chi-square test at *P* < 0.05. **(D)** Function of transferase activity involved in 4313 co-expressed DEGs versus the predicted protein-coding genes from the whole genome. Asterisk * indicate significant differences *P* < 0.05 by a Pearson chi-square test. **(E)** Function of response to stress involved in 4313 co-expressed DEGs versus the predicted protein-coding genes from the whole genome. Asterisk * indicate significant differences *P* < 0.05 by a Pearson chi-square test. **(F)** Function of catalytic activity which acting on a protein involved in 4313 co-expressed DEGs versus the predicted protein-coding genes from the whole genome. Asterisk * indicate significant differences *P* < 0.05 by a Pearson chi-square test

To further distinguish and analyze the function of genes in response to *V. dahliae* infection, DEGs were divided into two groups as shown in the Venn diagram in Fig. 2B. One group continuously expressed (co-expressed) DEGs at 6, 12, 24, 48, and 72 hpi, including 4313 genes, whereas the other group specifically expressed DEGs at 6, 12, 24, 48, and 72 hpi, encompassing 913, 681, 586, 3151, and 1150 genes, respectively. The co-expressed DEGs indicated that these genes responded steadily to *V. dahliae* infection by altering their expression levels. The expression levels of these 4313 genes at each of the five stages were hierarchically clustered using FPKM values (Fig. 4A). The expression patterns of 4313 genes were consistent among the five stages and could be classified into two groups: steadily up-regulated and steadily down-regulated (Fig. 4A). KEGG analysis showed that 4313 genes matched 101 pathway accessions (Table S6). Of these pathway accessions, 1552 co-expressed genes matched in the biosynthesis of secondary metabolites, 297 genes matched in phenylpropanoid biosynthesis, and 100 genes matched in pyruvate metabolism, which shares defense-related functions (Fig. 4B). In addition, GO analysis showed that the enrichment of co-expressed DEGs was high for transferase activity, binding, oxidation reduction, and phosphorus metabolism (*P* < 0.05) (Table S7 and Fig. 4C). Transferase function and activity in stress response have been reported to be involved in resistance and were further analyzed. Nine items involved in transferase activity (GO:0016740), transfer of glycosyl groups (GO:0016757), transfer of phosphorus-containing groups (GO:0016772), transfer of sulfur-containing groups (GO:0016782), and ubiquitin-like protein transferase activity (GO:0019787) were significantly enriched in the co-expressed DEGs (*P* < 0.05) (Fig. 4D). Furthermore, transferring glycosyl (GO:0016757) and phosphorus-containing groups (GO:0016772) occupied a large proportion of transferase activity (> 70%), whereas ubiquitin-like protein transferase activity (GO:0019787) was more enriched by co-expressed DEGs (7.08%) than by coding genes in the entire genome (3.46%) (Fig. 4D). Response to stress (GO:0006950), defense response (GO:00006952), and response to oxidative stress (GO:0006979) were significantly enriched in the co-expressed DEGs, accounting for 32.78% and 50.81%, respectively (*P* < 0.05) (Fig. 4E). As for the catalytic activity of proteins (GO:0140096), co-expressed DEGs were significantly enriched in protein kinase activity (GO:0004672) and ubiquitin-like protein transferase activity (GO:0019787) (Fig. 4F). Protein kinase activity accounted for the majority of this (75.94%), whereas the proportion of ubiquitin-like protein transferase activity enriched by co-expressed DEGs was higher than that of the coding genes in the whole genome (Fig. 4F). Together, these results show that the continuous function of the ZZM2 defense against *V. dahliae* stimulates transferase and catalytic activity.

### Functional analysis of specific-expressed DEGs in ZZM2 response to *V. dahliae* infection

**Fig. 5:**
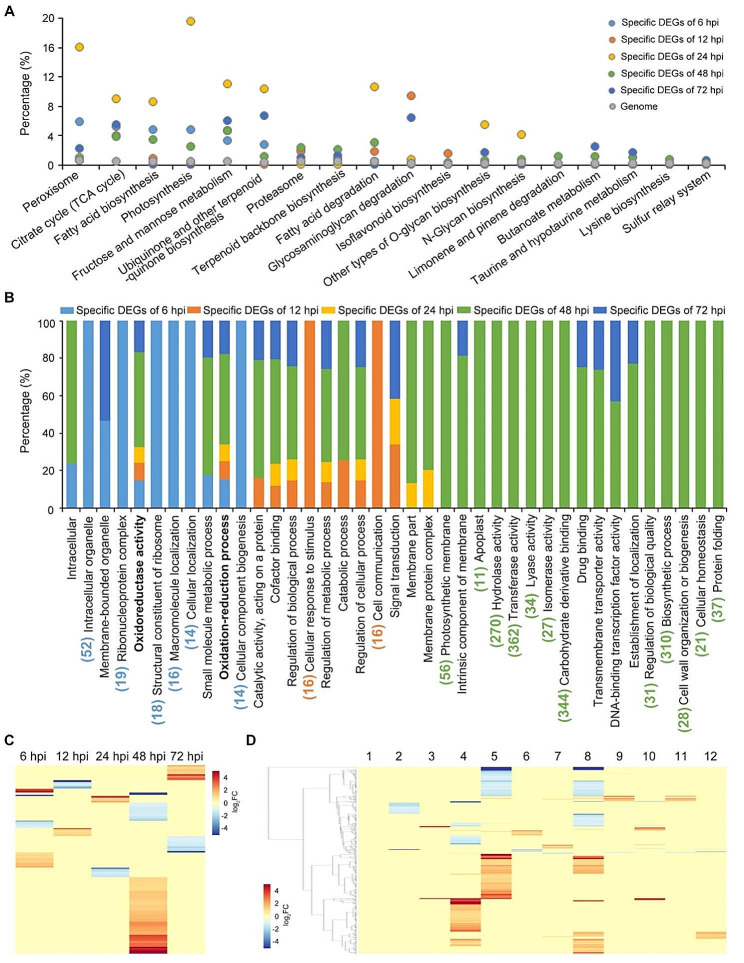
Functional annotation of the specific DEGs in cotton cv. Zhongzhimian No.2 (ZZM2) during *Verticillium dahliae* inoculation. (**A**) The potential pathways were predicted by the KEGG database specific DEGs at 6 hpi, 12 hpi, 24 hpi, 48 hpi, and 72 hpi respectively versus the predicted protein-coding genes from the whole genome. (**B**) Gene ontology (GO) annotation predicted specific DEGs at 6 hpi, 12 hpi, 24 hpi, 48 hpi, and 72 hpi respectively, and significant enrichment was determined by a Pearson chi-square test at *P* < 0.05. The number showed the number of DEGs enriched in the item, including the blue letters represented the number of DEGs only enriched at 6 hpi, the orange letters indicated the number of DEGs only enriched at 12 hpi, and the green letters showed the number of DEGs only enriched at 48 hpi. The bold letters mean the function items which were enriched at all of time stages. (**C**) Heatmap represented the time course expression profiles of specific DEGs annotated in ZZM2 at 6 hpi, 12 hpi, 24 hpi, 48 hpi, and 72 hpi respectively, involved in oxidoreductase activity and oxidation-reduction process, which was performed by FPKM values of all genes. (**D**) Heatmap represented the 12 function items, which only enriched at specific DEGs at 48 hpi, expression profiles performed by FPKM values of all genes

KEGG and GO analyses were performed to survey specific DEGs on the potential function of ZZM2 in defense against *V. dahliae* at different time points, at 6 (913), 12 (681), 24 (586), 48 (3151), and 72 hpi (1150). KEGG analysis matched specific DEGs at each stage to 136 pathway accessions (Table S8). Pathways matched by specific DEGs differed at each stage (Table S8 and Fig. 5A). Among these pathway accessions, peroxisomes, tricarboxylic acid cycle (TCA cycle), and fructose and mannose metabolism were matched by genes from all time points (Fig. 5A). Similar results were obtained from the GO analysis, with a total of 39 predicted functional items, and only two were enriched by specific DEGs in all five time points: oxidoreductase activity and oxidation-reduction process (*P* < 0.05) (Table S9 and Fig. 5B). The expression levels of genes involved in oxidoreductase activity and oxidation-reduction at each time point were hierarchically clustered using FPKM values (Fig. 5C). Additionally, several GO annotations were enriched for genes at specific time points. For example, DEGs at 6 hpi were enriched in intracellular organelles, functions of ribosomes, localization, and cellular component biogenesis; DEGs at 12 hpi were enriched in cellular response to stimuli and cell communication; and DEGs at 48 hpi were enriched in hydrolase, transferase, lyase, and isomerase activity, photosynthesis and biosynthetic processes, cell wall organization or biogenesis, and cellular homeostasis (Fig. 5B). The expression levels of these genes at 48 hpi were hierarchically clustered using FPKM values (Fig. 5D). ZZM2’s defense against *V. dahliae* relies on different genes at different time points, and 48 hpi is an important time point for the ZZM2 response to *V. dahliae* inoculation.

### ZZM2 regulated the function of the redox process to defend against *V. dahliae*

**Fig. 6:**
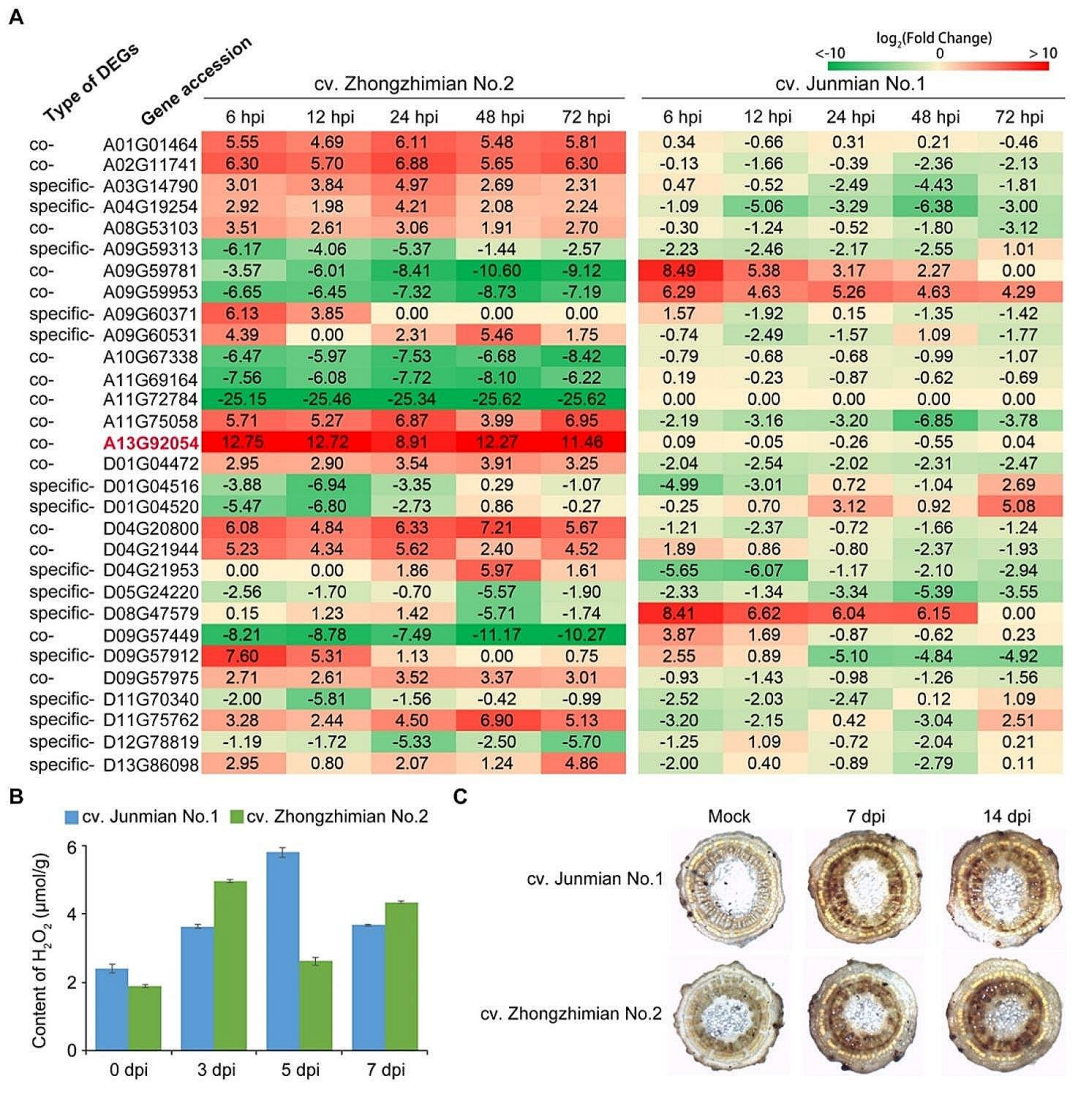
Function of redox process in cv. Zhongzhimian No.2 (ZZM2) to defense against *Verticillium dahliae*. (**A**) Expression patterns of 30 genes involved in oxidoreductase activity (GO:0016491) and oxidation-reduction process (GO:0055114) in resistant ZZM2 and susceptible J1 after inoculation with *V. dahliae* strain Vd991, detecting at 6 hpi, 12 hpi, 24 hpi, 48 hpi, and 72 hpi. (**B**) The content of H_2_O_2_ determined in roots of resistant ZZM2 and susceptible J1 after Vd991 inoculation at 3 days post inoculation (dpi), 5 dpi, and 7 dpi. (**C**) Detection of ROS-inducing activities in stems of resistant ZZM2 and susceptible J1 after Vd991 inoculation at 7 dpi and 14 dpi

Functional analysis showed that DEGs that responded to *V. dahliae* strain Vd991 infection were mainly enriched in functions related to redox processes, including peroxisomes, oxidoreductase activity, and oxidation-reduction process. In particular, oxidoreductase activity (GO:0016491) and oxidation-reduction process (GO:0055114) were both significantly enriched in co-expressed and specific DEGs at each time point (Figs. 4C and 5B). Thus, genes involved in oxidoreductase activity (GO:0016491) and oxidation-reduction process (GO:0055114) were selected for further study. A total of 329 co-expressed DEGs and 488 specific-expressed DEGs were identified (Table S10). To further examine the role of these genes in ZZM2, 30 genes were enriched in oxidoreductase activity (GO:0016491) and oxidation-reduction process (GO:0055114), and their expression patterns were significantly up- or down-regulated in ZZM2. Most genes were up-regulated in the resistant cultivar ZZM2 but were down-regulated or had no change in the susceptible cultivar J1 (Fig. 6A). Meanwhile, several genes were down-regulated in the resistant cultivar ZZM2 but were up-regulated or had no change in the susceptible cultivar J1 (Fig. 6A). The results showed that the expression of genes was regulated by Vd991 infection, and that these genes play a role in VW resistance via the redox process. Furthermore, H_2_O_2_ was detected in the cotton roots after Vd991 infection at 3, 5, and 7 dpi. The results showed that the H_2_O_2_ content in ZZM2 was higher than that in J1 at 3 dpi, and that the H_2_O_2_ content in J1 increased and was higher than that in ZZM2 at 5 dpi (Fig. 6B). In addition, ROS were detected by DAB staining at 7 and 14 dpi, and the results showed that ZZM2 was protected against Vd991 infection by increasing ROS levels (Fig. 6C). Therefore, the redox process plays an important role in ZZM2 in the defense against *V. dahliae*.

### Silencing of *COX1* repressed ZZM2 resistance to *V. dahliae* infection

**Fig. 7:**
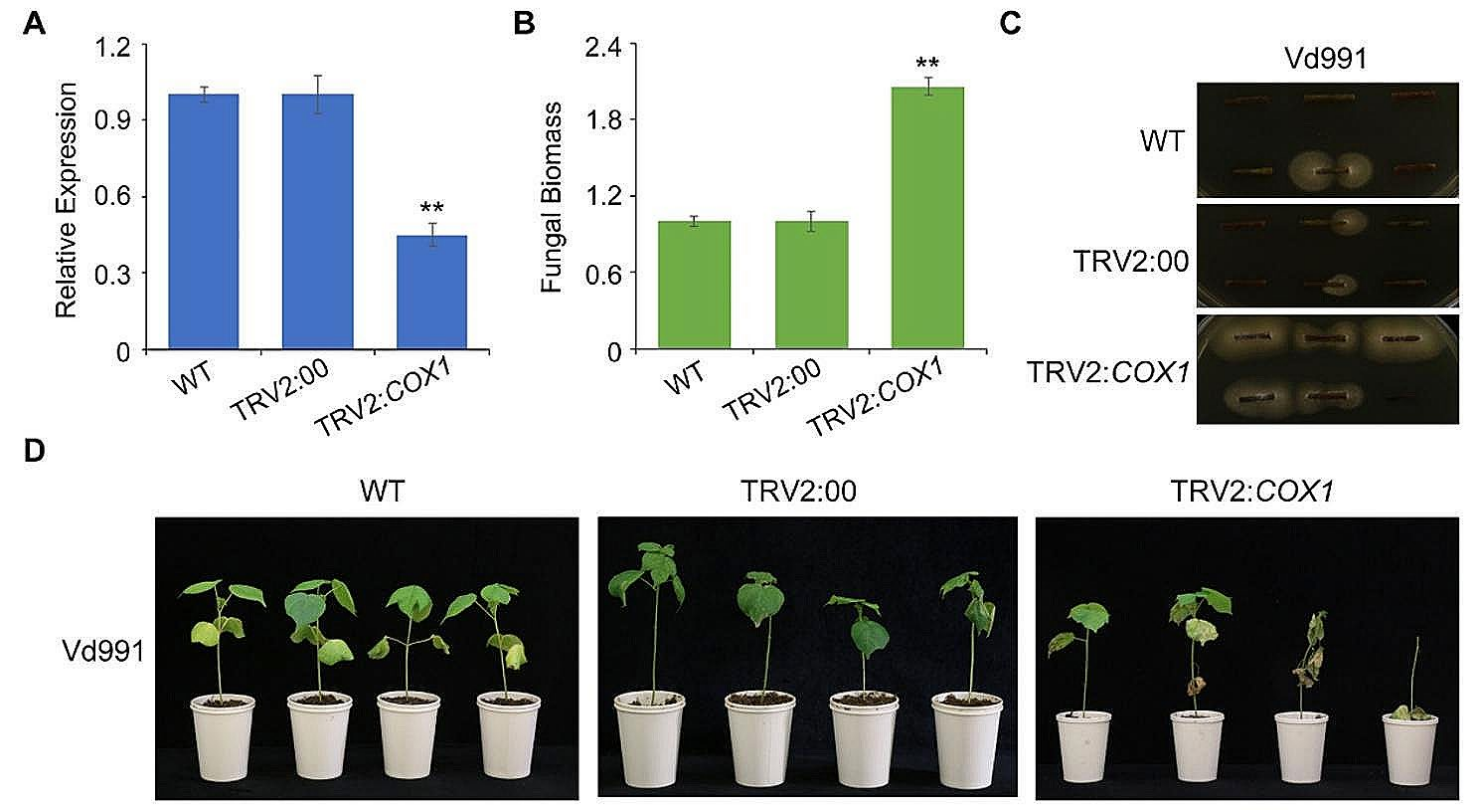
Silencing of the gene of *cytochrome c oxidase* (*COX1*) repressed the cv. Zhongzhimian No.2 (ZZM2) resistance to *Verticillium dahliae*. (**A**) The silencing efficiency of *COX1* was determined by RTqPCR analysis. The cotton *GhUbiquitin* gene was used as an endogenous control. The relative gene expression level in ZZM2 without infiltration were assigned a value of 1. Error bars indicate ± SD of three biological replicates, with each measured in triplicate. Asterisk ** indicate significant differences (*P* < 0.01). (**B**) The fungal biomass of silencing *COX1* in ZZM2 and infiltrating with empty vector pTRV2 (TRV2:00) detected by amplification of *V. dahliae* elongation factor *VdEF-1α* normalized by the cotton *18 S* gene through quantitative PCR (qPCR). The fungal biomass of wildtype (WT) ZZM2 was assigned a value of 1. Error bars indicate ± SD of three biological replicates, with each measured in triplicate. Asterisk ** indicate significant differences (*P* < 0.01). (**C**) *V. dahliae* recovery assay. Number of stem sections from which fungus grew described extent of fungal colonization. Photos were taken at 7 days after plating. Three biological replicates were conducted for analysis. (**D**) Silencing of *COX1* in ZZM2 by virusinduced gene silencing (VIGS) repressed ZZM2 resistance to *V. dahliae*. 3weeksold ZZM2 plants were used for VIGS, and seedlings infiltrated after 14 days were inoculated with *V. dahliae*. Experiments consisted of three replicates of 12 plants each arranged in a complete random block design. The Verticillium wilt phenotypes of wilting leaves and vascular discoloration were photographed 3–4 weeks after inoculation. Infiltration with the empty vector pTRV2 (TRV2:00) served as a control

The expression of *A13G92054* was significantly up-regulated in the resistant cultivar ZZM2, whereas no significant change was observed in the susceptible cultivar J1 (Fig. 6A). Therefore, *A13G92054* (*COX1*) was selected for further analysis. The *COX1* gene encodes cytochrome c oxidase and was up-regulated in ZZM2 cells after *V. dahliae* inoculation. Furthermore, we performed tobacco rattle virus (TRV)-based virus-induced gene silencing (VIGS) to assess the function of the *COX1* gene in ZZM2. The ratio of *COX1* silencing was quantified by RT-qPCR, and the results indicated that *COX1* expression was significantly decreased with more than 50% silencing ratio (Fig. 7A). *COX1*-silenced cotton, wild-type ZZM2, and control groups injected with TRV2::00 were challenged with Vd991. Stronger symptoms of VW were observed when *COX1* was silenced compared to those in the wild-type and control groups (Fig. 7D). The fungal biomass of *COX1*-silenced cotton tissue was significantly higher than that of wild-type tissues (Fig. 7B). In addition, fungal recovery showed that colonies thrived from *COX1*-silenced cotton tissues, while there was minimal growth observed from wild-type tissues (Fig. 7C).Results showed that *COX1*-silenced cotton tissues were more infected than the wild-type. Together, silencing the cytochrome c oxidase *COX1* significantly reduced ZZM2 VW resistance via a redox process.

## Discussion

*G. hirsutum* accounts for over 95% of cultivated cotton worldwide, and the used cultivars are typically susceptible to VW [34]. However, *G. hirsutum* cultivar ZZM2 possesses high yield and VW resistance. Therefore, identifying the mechanism of ZZM2 defense against VW is vital for breeding VW-resistant cotton cultivars. We previously assembled the whole genome of ZZM2 and showed that its secretome plays an important role in VW resistance [1, 26]. In this study, we constructed a gene network for ZZM2 in response to *V. dahliae* infection. Many DEGs in ZZM2 were identified during *V. dahliae* infection (Fig. 2). DEGs were identified as hydrolases, transferases, oxidoreductases, and genes involved in the biosynthesis of resistance-related compounds, such as starch and sucrose metabolism, and terpenoid-quinone biosynthesis (Fig. 3). Furthermore, these DEGs were divided into continuously expressed DEGs, which indicated a sustained response to *V. dahliae*, and DEGs specifically-expressed at each time point. These DEGs were predicted to play a role in the oxidation-reduction process and to act as oxidoreductases activity (Figs. 4 and 5). Genes related to the redox process were differentially expressed in ZZM2 after *V. dahliae* infection, and *COX1* silencing significantly reduced VW resistance in ZZM2 (Figs. 6 and 7). In addition, the H_2_O_2_ content in cotton roots was higher in ZZM2 than in the susceptible cultivar J1 after Vd991 infection (Fig. 6).

*G. hirsutum* cultivar ZZM2 showed excellent VW resistance and was used as a VW control in the regional experiments. ZZM2 was primarily used to identify the mechanism of action of VW resistance in cotton, and several genes have been shown to play important roles in VW resistance. These include the TIR-NBS-LRR gene *GhDSC1* [35], ribosomal protein GhRPS6 [36], respiratory burst oxidase homolog protein D GhRbohD [37], DUF668 (domain of unknown function 668) gene family [38], GhmiR395, and GhmiR165 [39]. Nevertheless, the molecular function, mechanism and gene regulatory network of ZZM2 defense against VW remain unclear. A previous study showed that *V. dahliae* enters the xylem vessels of the root after 48 h and then spreads to the neighboring xylem to sporulate [2]. Initial sporulation in the roots results in the rapid accumulation of fungal biomass, and plant defense responses can repress fungal proliferation and eliminate the fungus [2]. Thus, we identified DEGs in ZZM2 in response to *V. dahliae* infection from 6 to 72 hpi. Thousands of DEGs were identified at each time point and the maximum number of DEGs was observed at 48 hpi (Fig. 2A). The results showed that ZZM2 triggered a strong response at 48 h, which was identified as the critical time point. A total of 4313 DEGs were continuously expressed at all time points, and several DEGs were specifically expressed at 6, 12, 24, 48, and 72 hpi (Fig. 2B). Therefore, ZZM2 stimulated resistance mechanisms as soon as *V. dahliae* infection occurred, particularly 48 h after inoculation, with the highest number of DEGs against *V. dahliae* infection. Notably, several VW resistance genes were identified in the DEGs, including *GhDSC1*, *GhRPS6*, and *GhRbohD.* In addition, functional characterization predicted using these DEGs showed significant enrichment in defense responses (oxidoreductase activity, response to stress), cell wall strengthening (starch and sucrose metabolism), and the corresponding components involved in resistance (ascorbate and aldarate metabolism, flavonoid, and terpenoid quinone biosynthesis) (Fig. 3). For deep functional characterization analysis, the DEGs were divided into continuously- and specifically-expressed genes, indicating genes that were expressed steadily and genes that were expressed at specific time points, respectively. Continuously expressed DEGs were significantly enriched in genes involved in defense response (transferase activity, catalytic activity, and oxidoreductase activity), regulation of metabolic process (biosynthesis of secondary metabolites), and resistance-related process (plant hormone signaling, MAPK signaling, phenylpropanoid biosynthesis, and pyruvate metabolism) (Fig. 4). However, the functional characterization of specific DEGs showed apparent differences. For example, the specific DEGs at 6 hpi were mainly enriched in cellular components (intracellular organelles, structure of ribosome, cellular localization, and component biogenesis); DEGs specifically expressed at 12 hpi were primarily enriched in cellular response to stimulus and cell communication; DEGs specifically expressed at 24 hpi were highly enriched in energy metabolism (TCA cycle, photosynthesis, peroxisome); DEGs specifically expressed at 48 hpi were mainly enriched in kinase activity (hydrolase activity, transferase activity, lyase activity, and isomerase activity) (Fig. 5).

Two types of defense responses are triggered by the detection of different molecules secreted by pathogens: PTI and ETI. PTI induces ROS production, MAPK activation, callose deposition in the cell wall, and synthesis of pathogenesis-related proteins [12–14]. ROS are important signaling molecules that play significant roles in plant development, signal transduction, and environmental stress responses [40, 41]. H_2_O_2_ is the major form of ROS in plants and is mainly produced in peroxisomes, chloroplasts, and mitochondria [42]. In addition, the high H_2_O_2_ content in the apoplast, which is the extracellular space between the plasma membrane and cell wall, is toxic to plant cells [43]. ROS have a series of functions during plant defense against pathogens, including activation of defense gene expression, accumulation of phytoalexins, and internalization of pattern recognition receptors [44]. Moreover, ROS influence the production and signaling of phytohormones, and H_2_O_2_ functions in ABA signaling, which can activate MAPK and antioxidant enzymes [44]. In addition, ROS scavenging is important for VW resistance in cotton [7, 45]. In our study on the predicted function of genes in the ZZM2 response to *V. dahliae*, we observed that oxidoreductase activity (GO:0016491) and oxidation-reduction process genes (GO:0055114) were significantly enriched in all groups of DEGs (Figs. 3A and 4C, and 5B). In addition, the expression of genes involved in oxidoreductase activity and the oxidation-reduction process was associated with VW resistance because of the opposite expression patterns between ZZM2 and J1 (Fig. 6A). Furthermore, the levels of H_2_O_2_ and ROS were higher in the resistant cultivar ZZM2 than in the susceptible cultivar J1 (Fig. 6B and C). Overall, these results suggest that the defense of cotton ZZM2 against VW is primarily based on ROS accumulation.

Many genes have been found to function as important regulators of VW resistance in cotton via high-throughput sequencing and bioinformatics analyses, such as comparative proteomic analysis [7], small RNA sequencing (sRNA-seq) [11], specific-locus amplified fragment sequencing (SLAF-seq) [8], and genome-wide association studies (GWAS) [10, 17]. Transcriptome sequencing is suitable for revealing the molecular mechanisms of particular biological processes because it focuses on gene expression and transcriptional regulation and has been widely used to identify vital signaling pathways or resistance-related genes in response to VW in cotton. In this study, we predicted several candidate genes for VW resistance in the redox process based on bioinformatics-driven approaches using transcriptome sequencing of the VW-resistant cotton cultivar ZZM2 and the susceptible cultivar J1 (Fig. 6). The expression of *COX1* was significantly up-regulated in the resistant cultivar ZZM2, whereas no significant changes were observed in the susceptible cultivar J1. In addition, *COX1* silencing in ZZM2 repressed the resistance to *V. dahliae* (Fig. 7). Nevertheless, the functional mechanism of *COX1* needs to be elucidated in future studies and the VW-resistant functional mechanism of ZZM2 requires further research.

## Conclusions

In conclusion, our study confirmed the VW resistance of ZZM2, and conducted transcriptome sequencing to discover the DEGs in ZZM2 response to *V. dahliae* to reveal the gene regulatory networks of ZZM2 in defense against VW. The functional enrichment of DEGs related to resistance, such as biological process of flavonoid and terpenoid quinone biosynthesis, plant hormone signal, MAPK signaling, phenylpropanoid biosynthesis, pyruvate metabolism, and oxidation-reduction process. The expression of DEGs in ZZM2 enriched in oxidoreductase activity and oxidation-reduction process was opposite to those observed in the susceptible cultivar J1, and the production of ROS in ZZM2 was also significantly higher than in J1. Furthermore, gene silencing of *COX1* involved in oxidation-reduction process in ZZM2 increased susceptibility to *V. dahliae*. Overall, our findings demonstrated that the *G. hirsutum* cultivar Zhongzhimian No.2 response to *V. dahliae* via resistant related processes, especially the oxidation-reduction process. This contributes to a deeper understanding of the mechanisms regulating cultivar ZZM2 defense against VW.

### Electronic supplementary material

Below is the link to the electronic supplementary material.

Supplementary Material 1

Supplementary Material 2

Supplementary Material 3

Supplementary Material 4

Supplementary Material 5

Supplementary Material 6

Supplementary Material 7

Supplementary Material 8

Supplementary Material 9

Supplementary Material 10

## Data Availability

The data presented in this article have been deposited in the National Center for Biotechnology Information (NCBI) Sequence Read Archive (http://www.ncbi.nlm.nih.gov/sra/, accession number is PRJNA846595). The datasets generated for this research are available on request to the corresponding author.

## References

[1] Li R, Zhang YJ, Ma XY, Li SK, Klosterman SJ, Chen JY, Subbarao KV, Dai XF (2023). Genome resource for the Verticillium wilt resistant *Gossypium hirsutum* Cultivar Zhongzhimian 2. Mol Plant Microbe Interact.

[2] Fradin EF, Thomma BP (2006). Physiology and molecular aspects of Verticillium wilt diseases caused by *V. Dahliae* and *V. albo-atrum*. Mol Plant Pathol.

[3] Wang Y, Liang C, Wu S, Zhang X, Tang J, Jian G, Jiao G, Li F, Chu C (2006). Significant improvement of cotton Verticillium wilt resistance by manipulating the expression of gastrodia antifungal proteins. Mol Plant.

[4] Song R, Li J, Xie C, Jian W, Yang X (2020). An overview of the molecular genetics of plant resistance to the Verticillium wilt pathogen *verticillium dahliae*. Int J Mol Sci.

[5] Yang Z, Qanmber G, Wang Z, Yang ZE, Li FG (2020). Gossypium genomics: trends, scope, and utilization for cotton improvement. Trends Plant Sci.

[6] Xu J, Wang GL, Wang J, Li YQ, Tian LL, Wang XY, Guo WZ (2017). The lysin motif-containing proteins, Lyp1, Lyk7 and LysMe3, play important roles in chitin perception and defense against *Verticillium Dahliae* in cotton. BMC Plant Biol.

[7] Lu T, Zhu L, Liang Y, Wang F, Cao A, Xie S, Chen X, Shen H, Wang B, Hu M, Li R, Jin X, Li H (2022). Comparative proteomic analysis reveals the ascorbate peroxidase-mediated plant resistance to *Verticillium dahlia* in *Gossypium barbadense*. Front Plant Sci.

[8] Li TG, Ma XF, Li NY, Zhou L, Liu Z, Han HY, Gui YJ, Bao YM, Chen JY, Dai XF (2017). Genome-wide association study discovered candidate genes of Verticillium wilt resistance in upland cotton (*Gossypium hirsutum L*). Plant Biotechnol J.

[9] Zhang Y, Chen B, Sun ZW, Liu ZW, Cui YR, Ke HF, Wang ZC, Wu LQ, Zhang GY, Wang GN, Li ZK, Yang J, Wu JH, Shi RK, Liu S, Wang XF, Ma ZY (2021). A large-scale genomic association analysis identifies a fragment in Dt11 chromosome conferring cotton Verticillium wilt resistance. Plant Biotechnol J.

[10] Chen B, Zhang Y, Sun Z, Liu Z, Zhang D, Yang J, Wang G, Wu J, Ke H, Meng C, Wu L, Yan Y, Cui Y, Li Z, Wu L, Zhang G, Wang X, Ma Z (2021). Tissue-specific expression of *GhnsLTPs* identified via GWAS sophisticatedly coordinates disease and insect resistance by regulating metabolic flux redirection in cotton. Plant J.

[11] Hu G, Hao M, Wang L, Liu J, Zhang Z, Tang Y, Peng Q, Yang Z, Wu J (2020). The cotton miR477-CBP60A module participates in plant defense against *Verticillium dahlia*. Mol Plant Microbe Interact.

[12] Dodds PN, Rathjen JP (2010). Plant immunity: towards an integrated view of plant-pathogen interactions. Nat Rev Genet.

[13] He Q, McLellan H, Boevink PC, Sadanandom A, Xie C, Birch PR, Tian Z (2015). U-box E3 ubiquitin ligase PUB17 acts in the nucleus to promote specific immune pathways triggered by *Phytophthora infestans*. J Exp Bot.

[14] Thomma BP, Nurnberger T, Joosten MH (2011). Of PAMPs and effectors: the blurred PTI-ETI dichotomy. Plant cell.

[15] Jones J, Dangl J (2006). The plant immune system. Nature.

[16] Boller T, He SY (2009). Innate immunity in plants: an arms race between pattern recognition receptors in plants and effectors in microbial pathogens. Science.

[17] Zhang L, Wu YJ, Yu YG, Zhang YH, Wei F, Zhu QH, Zhou JL, Zhao LH, Zhang YL, Feng ZL, Feng HJ, Sun J. Acetylation of GhCaM7 enhances cotton resistance to *Verticillium Dahliae*. Plant J. 2023; 22. 10.1111/tpj.16200 36948889

[18] Alariqi M, Ramadan M, Wang Q, Yang Z, Hui X, Nie X, Ahmed A, Chen Q, Wang Y, Zhu L, Zhang X, Jin S (2023). Cotton 4-coumarate-CoA ligase 3 enhanced plant resistance to *Verticillium Dahliae* by promoting jasmonic acid signaling-mediated vascular lignification and metabolic flux. Plant J.

[19] Zhao J, Xu JW, Wang YP, Liu JG, Dong CG, Zhao L, Ai NJ, Xu ZZ, Guo Q, Feng GL, Xu P, Cheng JL, Wang X, Wang J, Xiao SH (2022). Membrane localized *GbTMEM214s* participate in modulating cotton resistance to Verticillium wilt. Plants.

[20] Liu S, Sun R, Zhang X, Feng Z, Wei F, Zhao L, Zhang Y, Zhu L, Feng H, Zhu H (2020). Genome-wide analysis of OPR family genes in cotton identified a role for *GhOPR9* in *Verticillium dahlia* resistance. Genes.

[21] Chang B, Zhao L, Feng Z, Wei F, Zhang Y, Zhang Y, Huo P, Cheng Y, Zhou J, Feng H (2023). Galactosyltransferase GhRFS6 interacting with GhOPR9 involved in defense against Verticillium wilt in cotton. Plant Sci.

[22] Pei Y, Li X, Zhu Y, Ge X, Sun Y, Liu N, Jia Y, Li F, Hou Y (2019). *GhABP19*, a novel germin-like protein from *Gossypium hirsutum*, plays an important role in the regulation of resistance to Verticillium and Fusarium wilt pathogens. Front Plant Sci.

[23] Qin T, Liu S, Zhang Z, Sun L, He X, Lindsey K, Zhu L, Zhang X (2019). *GhCyP3* improves the resistance of cotton to *Verticillium Dahliae* by inhibiting the E3 ubiquitin ligase activity of *GhPUB17*. Plant Mol Biol.

[24] Xiao SH, Hu Q, Shen JL, Liu SM, Yang ZG, Chen K, Klosterman SJ, Javornik B, Zhang XL, Zhu LF (2021). *GhMYB4* downregulates lignin biosynthesis and enhances cotton resistance to *Verticillium Dahliae*. Plant Cell Rep.

[25] Ministry of Agriculture and Rural Affairs of the People’s Republic of China. 2021. http://www.moa.gov.cn.

[26] Li R, Ma XY, Zhang YJ, Zhang YJ, Zhu H, Shao SN, Zhang DD, Klosterman SJ, Dai XF, Subbarao KV, Chen JY (2023). Genome-wide identification and analysis of a cotton secretome reveals its role in resistance against *Verticillium Dahliae*. BMC Biol.

[27] Zhang Y, Wang XF, Yang S, Chi J, Zhang GL, Ma ZY (2011). Cloning and characterization of a Verticillium wilt resistance gene from *Gossypium barbadense* and functional analysis in *Arabidopsis thaliana*. Plant Cell Rep.

[28] Kim D, Pertea G, Trapnell C, Pimentel H, Kelley R, Salzberg SL (2013). TopHat2: accurate alignment of transcriptomes in the presence of insertions, deletions and gene fusions. Genome Biol.

[29] Langmead B, Salzberg SL (2012). Fast gappedread alignment with bowtie 2. Nat Methods.

[30] Trapnell C, Williams B, Pertea G, Mortazavi A, Kwan G, van Baren MJ, Steven LS, Barbara JW, Lior P. Transcript assembly and quantification by RNASeq reveals unannotated transcripts and isoform switching during cell differentiation. Nat Biotechnol. 2010; 28511–5. 10.1038/nbt.1621 PMC3146043 20436464

[31] Kanehisa M, Goto S (2000). KEGG: kyoto encyclopedia of genes and genomes. Nucleic Acids Res.

[32] Li TG, Zhang DD, Zhou L, Kong ZQ, Hussaini AS, Wang D, Li JJ, Short DPG, Dhar N, Klosterman SJ, Wang BL, Yin CM, Subbarao KV, Chen JY, Dai XF (2018). Genome-wide identification and functional analyses of the CRK gene family in cotton reveals *GbCRK18* confers Verticillium wilt resistance in *Gossypium barbadense*. Front Plant Sci.

[33] Gao XQ, Britt RC, Shan LB, He P (2011). *Agrobacterium*mediated virusinduced gene silencing assay in cotton. J Vis Exp.

[34] Pegg GF, Brady BL (2002). Verticillium wilts (Cromwell Press).

[35] Li TG, Wang BL, Yin CM, Zhang DD, Wang D, Song J, Zhou L, Kong ZQ, Klosterman SJ, Li JJ, Adamu S, Liu TL, Subbarao KV, Chen JY, Dai XF. (2019). The *Gossypium hirsutum* TIR-NBS-LRR gene *GhDSC1* mediates resistance against Verticillium wilt. Mol. Plant Pathol. 2019a; 20: 857–876. 10.1111/mpp.12797 PMC6637886 30957942

[36] Zhu DD, Zhang XY, Zhou JL, Wu YJ, Zhang XJ, Feng ZL, Wei F, Zhao LH, Zhang YL, Shi YQ, Feng HJ, Zhu HQ (2021). Genome-wide analysis of ribosomal protein *GhRPS6* and its role in cotton Verticillium wilt resistance. Int J Mol Sci.

[37] Huang W, Zhang Y, Zhou J, Wei F, Feng Z, Zhao L, Shi Y, Feng H, Zhu H (2021). The respiratory burst oxidase homolog protein D (*GhRbohD*) positively regulates the cotton resistance to *Verticillium Dahliae*. Int J Mol Sci.

[38] Zhao JY, Wang P, Gao WJ, Long YL, Wang YX, Geng SW, Su XN, Jiao Y, Chen QJ, Qu YY (2021). Genome-wide identification of the *DUF668* gene family in cotton and expression profiling analysis of *GhDUF668* in *Gossypium hirsutum* under adverse stress. BMC Genomics.

[39] Mei J, Wu Y, Niu Q, Miao M, Zhang D, Zhao Y, Cai F, Yu D, Ke L, Feng H, Sun Y (2022). Integrative analysis of expression profiles of mRNA and microRNA provides insights of cotton response to *Verticillium Dahliae*. Int J Mol Sci.

[40] Mittler R, Vanderauwera S, Gollery M, Van Breusegem F (2004). Reactive oxygen gene network of plants. Trends Plant Sci.

[41] Li HB, Qin YM, Pang Y, Song WQ, Mei WQ, Zhu YX (2007). A cotton ascorbate peroxidase is involved in hydrogen peroxide homeostasis during fiber cell development. New Phytol.

[42] Zhao Y, Yu H, Zhou JM, Smith SM, Li J (2020). Malate circulation: linking chloroplast metabolism to mitochondrial ROS. Trends Plant Sci.

[43] Smirnoff N, Arnaud D (2019). Hydrogen peroxide metabolism and functions in plants. New Phytol.

[44] Lu Y, Yao J (2018). Chloroplasts at the crossroad of photosynthesis, pathogen infection and plant defense. Int J Mol Sci.

[45] Li ZK, Chen B, Li XX, Wang JP, Zhang Y, Wang XF, Yan YY, Ke HF, Yang J, Wu JH, Wang GN, Zhang GY, Wu LQ, Wang XY, Ma ZY (2019). A newly identified cluster of glutathione S-transferase genes provides Verticillium wilt resistance in cotton. Plant J.

